# Optical coherence tomography parameters as prognostic factors for stereopsis after vitrectomy for unilateral epiretinal membrane: a cohort study

**DOI:** 10.1038/s41598-024-57203-x

**Published:** 2024-03-20

**Authors:** Simei Shen, Siyan Jin, Fuqiang Li, Jinsong Zhao

**Affiliations:** Department of Ophthalmology, The Second Hospital of Jilin University, Changchun, 130041 China

**Keywords:** Anatomy, Diseases, Health care, Medical research, Risk factors

## Abstract

This retrospective cohort study explored the relationship between monocular and interocular optical coherence tomography (OCT) parameters and stereopsis in 56 patients undergoing pars plana vitrectomy (PPV) for unilateral idiopathic epiretinal membrane (IERM). IERM impairs visual functions, with symptoms ranging from asymptomatic to severe impairment. Despite established surgical interventions, including PPV with membrane peeling, the impact on advanced three-dimensional visual functions such as stereopsis remains inadequately investigated. All subjects were assessed for stereopsis, visual acuity, and metamorphopsia, alongside spectral domain OCT parameters. These visual functions significantly improved 3-month postoperatively. Central retinal thickness at the fovea, parafovea, and perifovea (CFT, CRT-3 mm, and CRT-6 mm), ectopic inner foveal layer thickness, and retinal layer thickness notably decreased 1 week to 3 months after surgery. The interocular difference in OCT parameters between bilateral eyes was included as a parameter. Baseline CRT-3 mm difference and inner nuclear layer (INL) thickness were independently correlated with postoperative stereopsis on the Titmus Stereo Test, while baseline CRT-6 mm difference and INL thickness were independently related to stereopsis on the TNO stereotest. This study highlights the substantial enhancement in stereopsis post-IERM surgery, with both interocular and monocular OCT parameters independently influencing postoperative stereopsis. These findings underscore the importance of retinal microstructures in assessing and predicting stereopsis in IERM patients after vitrectomy.

## Introduction

The epiretinal membrane (ERM) is characterized by the proliferation of contractile avascular fibrocytes that gradually form a transparent membrane on the vitreoretinal macula surface^1^, occurring in 3.4–28.9% of the worldwide population, and the prevalence increases with age^2–8^. The formation of ERMs can be idiopathic or secondary to ocular diseases, including inflammatory diseases, trauma, tumors, intraocular surgery, or retinal detachment. Among these, idiopathic epiretinal membrane (IERM) is the most prevalent, accounting for approximately 67.7% of ERM and typically occurring in older patients^8^.

The symptoms of IERM range widely from asymptomatic to severe visual impairment, such as decreased best-corrected visual acuity (BCVA), metamorphopsia, and aniseikonia, contrast sensitivity, and among others^9^. Many researchers have suggested that these visual impairments result from macular thickening and retinal contraction in the affected eye. Pars plana vitrectomy (PPV) with membrane peeling is the optimal surgical intervention for restoring visual function by alleviating deformed retinal microstructures in patients with ERM^10–13^. In contrast, only a few studies have evaluated the impact of IERM on stereopsis, stating that stereopsis in unilateral IERM was constantly inferior than that in the same age group and showed partial recovery following PPV^14–18^. Despite the impact of BCVA, metamorphopsia, contrast sensitivity, and aniseikonia on stereopsis in IERM patients, the decline in stereopsis could also occur independently of these two-dimensional visual functions and significantly affect the visual quality of IERM patients^15,19^. Thus, it is imperative to conduct research on stereopsis, an advanced three-dimensional visual function, in the aftermath of IERM surgery.

As optical coherence tomography (OCT) can provide high-resolution retinal images, thereby presenting near-histologic details, various OCT-based macula measurements have been proposed and identified as relevant factors for BCVA improvement, metamorphopsia reduction, and reduced aniseikonia following surgery in IERM eyes^10,13,20,21^. Although, there is a scarcity of research exploring the correlation between anatomical disruptions and stereopsis in IERM. Currently, only one study has reported that the Titmus Stereo Test (TST) and TNO stereotest (TNO) stereopsis were associated with a number of OCT-related parameters preoperatively and postoperatively, such as central foveal thickness (CFT), central retinal thickness at the parafovea (CRT-3 mm), inner nuclear layer (INL) thickness, macular volume, outer nuclear layer-outer plexiform layer thickness, Ganglion cell layer thickness, and the like ^22^. Besides, in most IERM cases, binocular misregistration of retinal mosaics due to foveal displacement ^23,24^, potentially leading to abnormal retinal correspondence and consequent stereopsis impairment. These instances suggested the involvement of differing macular structures between affected and contralateral healthy eyes in influencing stereopsis, yet the direct relationship between anatomical variations in both the operated eye and its counterpart and stereopsis in IERM patients remains unknown.

This study aimed to determine the prognostic effects of binocular and monocular retinal microstructures for TST and TNO stereopsis in unilateral IERM patients who underwent PPV with ERM peeling. Secondary aim was to investigate the prognostic effects of BCVA and metamorphopsia for stereopsis.

## Results

### Clinical features of unilateral idiophathic epiretinal membrane before surgery

Table 1. includes the demographic statistics and comparisons of the clinical characteristics between bilateral eyes. Fifty-six patients (44 women and 12 men) with an average age of 64.89 ± 1.03 years (mean ± standard deviation) were enrolled. All patients complained of distorted or blurred vision, or reduced visual acuity. Duration ranged from 1 to 72 months, with a mean of 16.43 ± 2.25 months, and duration of exacerbation ranged from 0 to 10 months, with a mean of 2.29 ± 0.36 months. All participants underwent standard 25G three-port PPV with ERM peeling surgery, and 16 patients underwent concurrent cataract surgery. Two cases presented peripheral pinpoint holes and were filled with sterilized air, and the remaining 54 cases were all filled with balanced salt solution. All patients were followed up for at least 3 months, and a flat retina was observed during each postoperative slit-lamp examination. One patient had small blood accumulation in the anterior chamber on postoperative day 2. Within 1 week after surgery, both the anterior chamber blood and the intraocular gas were reabsorbed. Differential statistical analysis reveals that the BCVA of the affected eye is significantly inferior to that of the healthy eye. Measures of CFT, CRT-3 mm, perifoveal retinal thickness (CRT-6 mm), ganglion cell layer-inner plexiform layer (GCL-IPL) thickness, INL thickness, outer nuclear layer (ONL) thickness were significantly higher in the affected eye. Furthermore, the affected eye presented no metamorphopsia and ectopic inner fovea layer (EIFL), and displayed integrated external limiting membrane (ELM), ellipsoid zone (EZ) and cost outer segment tip (COST) lines in the outer retina.

**Table 1:** Data at baseline of patients with unilateral idiopathic epiretinal membrane.

Indicator	Value		
Age (year) (range)	64.89 ± 1.03 (47–76)		
Gender (male/female) no. (%)	12/44 (78.57%/21.43%)		
Eye category (right/left) no. (%)	34/22 (60.71%/39.29%)		
Duration (month) (range)	16.43 ± 2.25 (1–72)		
Aggravated duration (month) (range)	2.29 ± 0.36 (0–10)		
Surgical procedure no. (%)			
Vitrectomy	40 (71.43%)		
Vitrectomy + IOL + BSS	14 (25%)		
Vitrectomy + IOL + sterilized air	2 (35.71%)		
Titmus stereo test value (log)	2.64 ± 0.08		
TNO stereotest value (log)	3.19 ± 0.06		

Indicator	Affected eye	Fellow eye	P
MV (°) (range)	0.286 ± 0.239 (0–0.9)	0	
MH (°) (range)	0.318 ± 0.482 (0–2.0)	0	
Phakic eye no. (%)	56(100%)	56(100%)	
Intraocular pressure (mmHg)	16.04 ± 0.40	16.40 ± 0.34	0.199
BCVA (logMAR)	0.56 ± 0.06	0.15 ± 0.03	< 0.0001**
CFT (μm)	459.04 ± 13.76	269.53 ± 5.12	< 0.0001**
CRT-3 mm (μm)	433.33 ± 10.67	334.57 ± 2.45	< 0.0001**
CRT-6 mm (μm)	352.01 ± 5.90	299.50 ± 2.87	< 0.0001**
EIFL thickness (μm)	154.50 ± 11.86	0	
GCL-IPL thickness (pixel)	14,484.79 ± 408.90	9462.46 ± 150.15	< 0.0001**
INL thickness (pixel)	7701.57 ± 325.71	4117.29 ± 121.81	< 0.0001**
ONL thickness (pixel)	12,301.96 ± 685.27	9120.77 ± 192.73	< 0.0001**
ELM disruption no. (%)	12 (21.43%)	0	
EZ disruption no. (%)	8 (14.29%)	0	
COST disruption no. (%)	26 (46.43%)	0	

Continuous values are presented as mean ± standard deviation; comparisons between two eyes were calculated by Wilcoxon signed-rank test.

Visual functions and microstructures significantly prior to the affected eye.

*IOL* surgery with lens extraction and intraocular lens implantation, *BSS* balanced salt solution, *IOP* intraocular pressure, *BCVA* best-corrected visual acuity, *LogMAR* logarithm of the minimum angle of resolution, *MV* vertical metamorphopsia, *MH* horizontal metamorphopsia, *CFT* central foveal thickness, *CRT-3 mm* central retinal thickness at the parafovea, *CRT-6 mm* central retinal thickness at the perifovea, *EIFL* ectopic inner fovea layer, *GCL-IPL* ganglion cell layer-inner plexiform layer, *INL* inner nuclear layer, *ONL* outer nuclear layer, *ELM* external limiting membrane, *EZ* ellipsoid zone, *COST* cone outer segment tip.

*P < 0.001; **P < 0.0001.

### Temporal changes of visual functions and OCT-related parameters in patients with ERM

**Figure 1:**
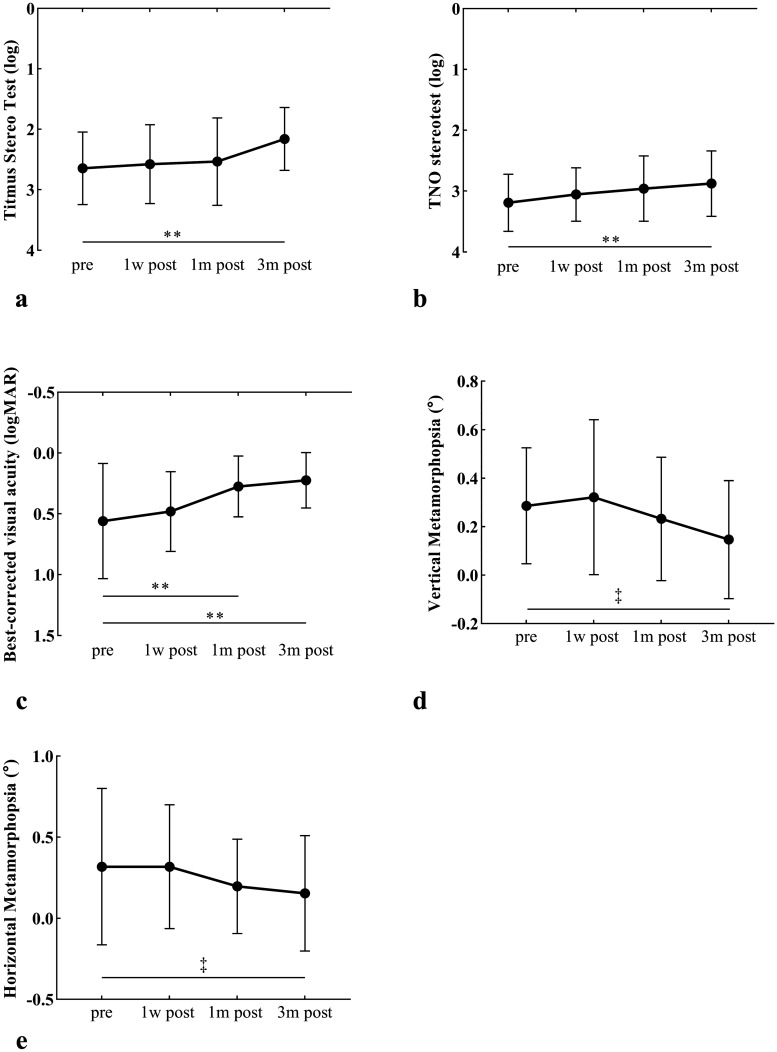
Temporal changes in visual function in patients with epiretinal membrane before and after surgery. Mean and standard deviation were shown in black dot and error bars. **(a)** Titmus stereo test value.** (b)** TNO stereotest value. **(c)** Best-corrected visual acuity. **(d)** Vertical metamorphopsia. **(e)** Horizontal metamorphopsia. Visual functions significantly improved after surgery. ^†^P < 0.05; ^‡^P < 0.01; *P < 0.001; **P < 0.0001.

Figure 1. shows temporal changes of visual functions. Vitrectomy considerably enhanced the mean TST value (log) to 2.159 ± 0.519 (P < 0.0001), promoted TNO valuestereopsis (log) to 2.873 ± 0.515 (P < 0.0001), dimmed MV to 0.146 ± 0.243 (P = 0.010), and weakened MH to 0.154 ± 0.355 (P = 0.001) 3 months postoperatively. The BCVA (logMAR) improved to 0.223 ± 0.228 at 1 month and 0.274 ± 0.249 at 3 months after surgery (both P < 0.0001). Temporal changes of OCT-related parameters were placed in Supplementary Fig. 1. CFT and CRT-6 mm significantly became thinner at 1 month and 3 months after surgery. The preoperative CRT-3 mm was prominently thicker than those measured at 1 week, 1 month, and 3 months postoperatively, and similar results were found for GCL-IPL, INL and ONL. Considerable thinner EIFL thickness was recorded at 1 month and 3 months after surgery. ELM returned to the original shape in all ERMs at 1 month postoperatively and significantly improved 1 week after vitrectomy. In contrast, vitrectomy minimally affected the disruption of the EZ and COST lines.

**Figure 2:**
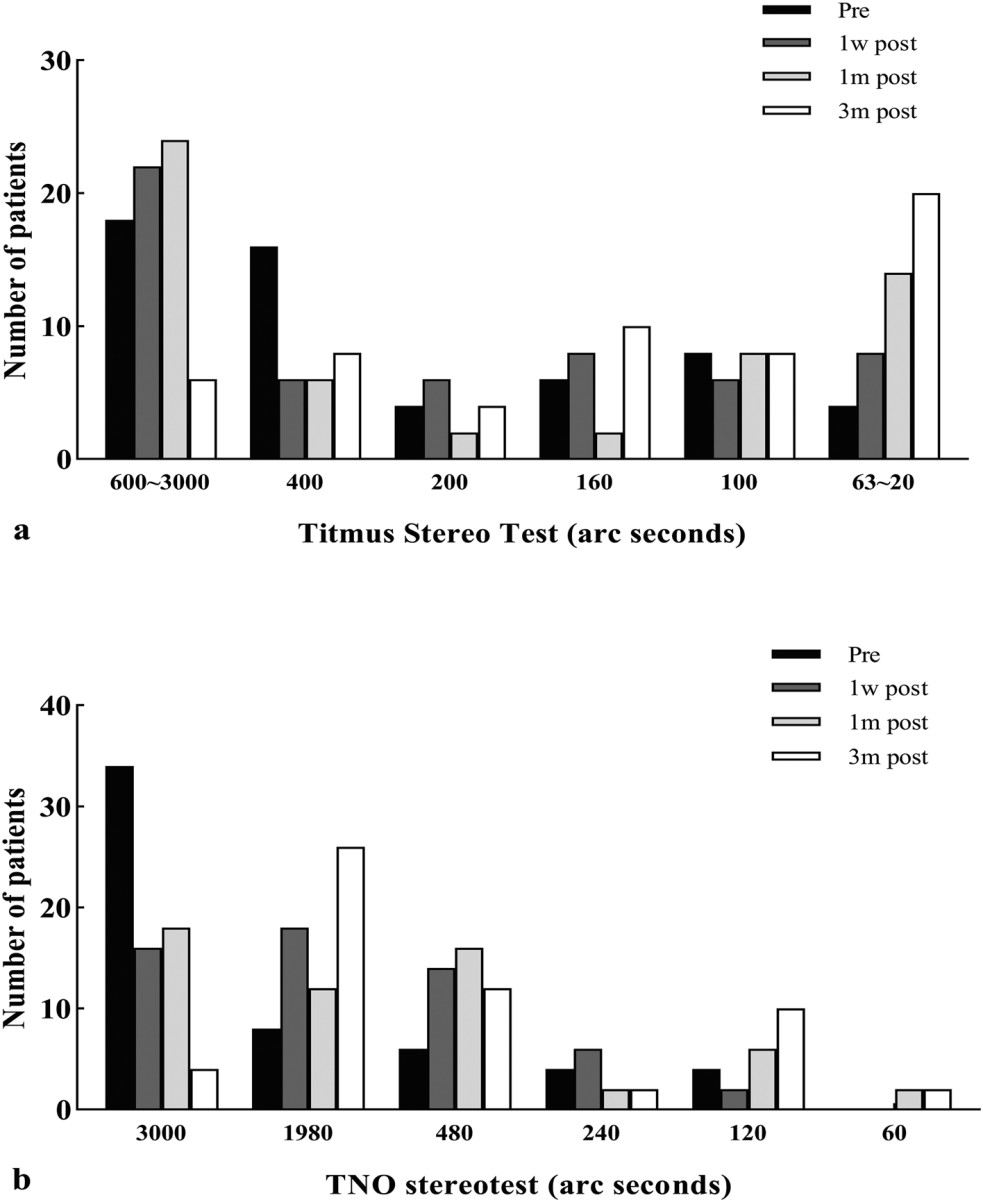
Stereoacuity for 56 patients with a unilateral IERM before and after surgery. **(a)** Histogram of the Titmus stereo test value, **(b)** histogram of the TNO stereotest value.

For most IERMs 3-month postoperatively, TNO value only reaches the level of partially raised icons, typically around the 1980" mark (Fig. 2a). In contrast, by three months post-surgery, the majority of patients had regained good TST value, within the range of 63" to 20" (Fig. 2b). The TST stereopsis with one eye covered were not superior to those with both eyes exposed for all patients at each point.

### Correlation between stereopsis and other parameters before and after surgery

The results of univariate correlation analysis by Spearman rank correlation were shown in Supplementary Table 1. and Supplementary Table 2. Before unilateral IERM surgery, preoperative TST value exhibited significant positive correlations with BCVA (P = 0.002), patient age (P = 0.003), CFT thickness (P = 0.007), CRT-3 mm thickness (P = 0.029), GCL-IPL thickness (P = 0.04), INL (P = 0.015), and ONL thickness (P = 0.047), while negatively correlated with CFT difference (P < 0.001), CRT-3 mm difference (P = 0.001), CRT-6 mmc difference (P = 0.05), GCL-IPL thickness difference (P = 0.036), INL thickness difference (P = 0.025), and ONL thickness difference (P = 0.017). TNO value at baseline had no relationship with BCVA or metamorphopsia, but exhibited a negative correlation with CFT difference (P = 0.005) and showed positive associations with patient age (P = 0.014), CFT (P = 0.009), and INL thickness (P = 0.016).

**Figure 3:**
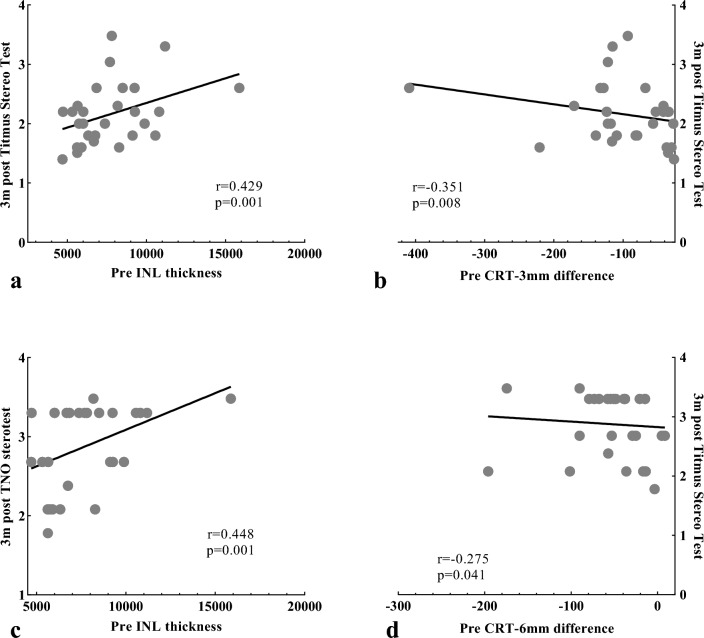
Correlation between postoperative stereopsis and preoperative OCT parameters in patients with ERM. **(a)** Postoperative Titmus Stereo Test value versus preoperative INL thickness. **(b)** Postoperative Titmus Stereo Test value versus preoperative CRT-3 mm difference. **(c)** Postoperative TNO stereotest value versus preoperative INL thickness. **(d)** Postoperative TNO stereotest value versus preoperative CRT-6 mm difference.

At three months after PPV, postoperative TST value was positively associated with age (P = 0.02), pre- TST value, pre- TNO value, 1-month post- BCVA, 3-month post- BCVA (P < 0.001, P < 0.001, P < 0.001, and P = 0.001, respectively), and postoperative measurements of CFT (P = 0.001), CRT-3 mm (P = 0.001), CRT-6 mm (P = 0.003), EIFL thickness (P = 0.007), GCL-IPL thickness (P = 0.012), and INL thickness (P = 0.015), negatively linked to CFT difference (P = 0.001), CRT-3 mm difference (P = 0.008), and INL thickness difference (P = 0.001), and preoperative CFT (P = 0.048), EIFL thickness (P = 0.019), INL thickness (P = 0.001, Fig. 3a), CFT difference (P = 0.001), CRT-3 mm difference (P = 0.008, Fig. 3b), INL thickness difference (P = 0.001). Postoperative TNO value at the three-month mark was not correlated with pre- TNO value, but displayed positive correlation BCVA, TST value, and MV at baseline (P = 0.040, P < 0.001, and P = 0.013, respectively) and 3-month after surgery (P < 0.001, P = 0.004, and P < 0.001, respectively), and postoperative CFT (P < 0.001), CRT-3 mm (P < 0.001), CRT-6 mm (P = 0.001), EIFL thickness (P = 0.001), GCL-IPL thickness (P = 0.027), INL thickness (P < 0.001), ONL thickness (P = 0.007), and EZ disruption (P = 0.001), and showed negative correlations with postoperative CFT difference (P = 0.001), CRT-3 mm difference (P < 0.001), CRT-6 mm difference (P = 0.041), GCL-IPL thickness difference (P = 0.011), and INL thickness difference (P = 0.019). Postoperative TNO value showed a significant correlation with preoperative TST value (P < 0.001), BCVA (P = 0.040), MV (P = 0.013), CFT (P = 0.007), CRT-3 mm (P < 0.001), CRT-6 mm (P = 0.011), EIFL thickness (P = 0.001), GCL-IPL thickness (P = 0.001), INL thickness (P = 0.001, Fig. 3c), CFT difference (P = 0.001), CRT-3 mm difference (P < 0.001), CRT-6 mm difference (P = 0.041, Fig. 3d), GCL-IPL difference (P = 0.011), INL difference (P = 0.019), and COST disruption (P = 0.007). Additionally, TST and TNO value, both preoperatively and three months postoperatively, showed no correlation with eye category, duration, aggravated duration, or surgical procedure.

### Independent factors for preoperative and postoperative stereopsis in ERM

The multiple stepwise linear regression analysis results for stereopsis are listed in Tables 2 and 3. Preoperative TST value was associated with preoperative CRT-3 mm (P = 0.002) and the variance between the affected eye and contralateral eye (P < 0.001), and the standardized partial regression coefficient (β) was −0.693 and −1.049, respectively. Preoperative TNO value was only independently associated with age (P = 0.004, β = 0.376). The postoperative 3-month CFT was independently linked to both TST value (P = 0.002, β = 0.404) and TNO value after surgery (P < 0.001, β = 0.633)..

**Table 2:** Correlation between preoperative stereopsis and OCT parameters in patients with IERM.

	β	P	F	Adjusted R^2^	Durbin-Watson
Preoperative Titmus stereo test value (log)					
Preoperative CRT-3 mm	−0.693	0.002	13.628	0.315	1.754
CFT difference	−1.049	< 0.001			
Postoperative Titmus stereo test value (log)					
Postoperative 3 months CFT	0.404	0.002	10.545	0.148	2.008
Preoperative TNO stereotest value (log)					
Age	0.376	0.004	8.864	0.125	2.286
Postoperative TNO stereotest value (log)					
Postoperative 3 months CFT	0.633	< 0.001	36.013	0.389	2.175

*CRT-3 mm* central retinal thickness at the parafovea, *CFT* central foveal thickness, *CFT difference* value obtained by subtracting the CFT of the operated eye from that of the contralateral healthy eye.

**Table 3:** Correlation between 3-month postoperative stereopsis and preoperative parameters in patients with unilateral IERM.

	β	P	F	Adjusted R^2^	Durbin-Watson
Postoperative 3 months Titmus stereo test value (log)					
Preoperative TST value	0.700	< 0.001	27.387	0.590	1.930
Preoperative INL thickness	0.673	< 0.001			
Difference of CRT-3 mm	0.508	0.002			
Postoperative 3 months TNO stereotest value (log)					
Preoperative TST value	0.468	< 0.001	12.914	0.464	2.191
Preoperative INL thickness	0.433	< 0.001			
Preoperative MV	0.334	0.003			
Difference of CRT-6 mm	0.355	0.004			

The ‘difference’ here refers to the value obtained by subtracting the retinal thickness of the operated eye from that of the contralateral healthy eye.

*INL* inner nuclear layer, *MV* vertical metamorphopsia, *CRT-3 mm* central retinal thickness at the parafovea, *CRT-6 mm* central retinal thickness at the perifovea.

Postoperative TST value was independently associated with preoperative TST value (P = 0.008, β = 0.7), INL thickness (P < 0.001, β = 0.673) and bilateral difference of CRT-3 mm (P = 0.002, β = 0.508). After all preoperative variables were considered, the analysis showed that those with predictive value for postoperative TNO value were TST value (P < 0.001, β = 0.468), INL thickness (p < 0.001, β = 0.433), MV (P = 0.003, β = 0.334), and bilateral difference of CRT-6 mm (P = 0.004, β = 0.355) at baseline.

## Discussion

The current finding revealed that 56 patients (56 eyes) diagnosed with unilateral IERM suffered from impaired stereopsis, decreased visual acuity, metamorphopsia, thickened retinal thickness, and chaotic microstructures. A positive response to both symptomatic distress and anatomical irregularities was observed in all subjects during the 3-month follow-up. Beyond preoperative visual functions (TST stereopsis and vertical metamorphopsia), our study identified preoperative microstructures, including the INL thickness and the disparity in central retinal thickness (CRT-3 mm and CRT-6 mm) between the affected eye and the contralateral eye, as potential predictive factors for postoperative stereopsis in patients undergoing unilateral IERM surgery. Notably, only one prior study from Japan has explored the association between OCT measurements of the affected eye and postoperative stereopsis after IERM surgery^22^. Our investigation marks the first exploration of this relationship in Chinese patients, and it is the initial study to delve into the connection between interocular anatomical differences and stereopsis in IERMs.

Spearman correlation analysis revealed consistent positive correlations between stereopsis and various optical coherence tomography (OCT) findings, both preoperatively and postoperatively. These findings encompassed metrics such as CFT, CRT-3 mm, CRT-6 mm, as well as inner and outer retinal layer thicknesses (GCL-IPL, INL, and ONL), and disruptions in the photoreceptor layer (EZ). Several researchers have observed that anatomical impairments affect stereopsis in macular pathologies like macular hole, age-related macular degeneration (AMD), rhegmatogenous retinal detachment (RRD), and central retinal vein occlusion (CRVO). These studies have predominantly been on the affected eye's anatomical structure. In comparison, our study employed somewhat different protocols, for that the disparity in binocular OCT parameters was included. The univariate analysis showed that TST and TNO values were significantly correlated with various binocular retinal differences before or after surgery, including CFT difference, CRT-3 mm difference, CRT-6 mmc difference, GCL-IPL thickness difference, INL thickness difference, ONL thickness difference, and EIFL thickness difference. The multiple regression analysis identified that preoperative difference in CRT-3 mm and disparity in CRT-6 mm of both two eyes, rather than individual eye measurements, were independent factors linked to improved postoperative TST and TNO stereopsis following unilateral IERM surgery. Possible explanation for this finding is that disruption of retinal elements may cause retinal misregistration, subsequently impairing binocular function ^23^. Hence, assessing differences in retinal thickness between both eyes, rather than focusing solely on the affected eye's retinal thickness, may serve as a more practical way to predict stereopsis outcomes.

While Okamoto previously established the prognostic independent impact of preoperative CRT-3 mm on postoperative TST value, our study delved deeper. We not only confirmed the independent predictive role of retinal thickness at the parafovea (CRT-3 mm) for postoperative TST value but also extended the findings to the significance correlation of retinal thickness at the perifoveal region (CRT-6 mm) with postoperative TNO value. According to previous studies, this discrepancy between TST value and TNO value might stem from difference in the size of their stimuli^22,25^. At the standard examination distance, the TST circles subtend a visual angle of 0.7° and 1 set of 4 circles subtends ~ 2.5°, and TNO stereo-target subtends a visual angle of 8.5°. The wider range of TNO stimuli might impact a broader retinal area. Therefore, CRT-3 mm correlated with TST value, whereas, broader CRT-6 mm region exhibited a correlation with TNO value. In addition, the parafoveal region's rod cells generate diffusible substances crucial for the survival of cone cells^26^, implying that the parafovea sustains the visual function of the macular area. Hence, the disturbed retina at the peripheral region around the fovea might affect the stereopsis.

According to recent researches, binocular stereopsis is greatly impacted by monocular impairment in BCVA in various retinal disorders, including CRVO^27,28^, RRD^25^,Macular hole^29^, and ERM^16^. Spearman correlation analysis in our results revealed a positive relationship between preoperative TST value and pre-BCVA, postoperative stereopsis and post-BCVA, as well as post-TNO value and pre-BCVA, which highlighted that is monocular visual acuity impairment closely linked to stereopsis. These aligns with studies simulating monocular visual acuity impaired in normal individuals ^30,31^. However, we observed a phenomenon wherein the enhancement of BCVA and stereopsis in IERM did not consistently coincide, an observation that may have been overlooked in previous studies^14–18,22,32^. Firstly, the temporal dynamics of BCVA and stereopsis were asynchronous: the mean BCVA showed improvement at 1-month postoperatively and reached a plateau at 3-month postoperatively, whereas TST and TNO stereopsis exhibited improvement only starting from 3 months postoperatively. Secondly, the extent of improvement was also asynchronous: for instance, in patient 11, BCVA (logMAR) improved from 0.7 to 0.2, while TST stereopsis remained constant at 3000" at each measurement, and TNO stereopsis only improved from 3000" to 1980". In another case, patient 27 experienced an improvement in TST stereopsis from 3000" preoperatively to 400", while BCVA remained stable 3 months postoperatively. Consequently, the Titmus and TNO stereopsis tests demonstrated the possible capacity to evaluate surgical outcomes independently of visual acuity. Therefore, further research is needed to explore the correlation between visual acuity and stereopsis in macular diseases.

The results of multiple regression analysis indicated that preoperative thinning INL was a determining factor for the reduction in both TST and TNO stereopsis following IERM surgery. Existing researches have elucidated that macular displacement leading to retinal misalignment is prevalent among IERM patients^23,24^, with false binocular parallax potentially impacting stereopsis. Specifically, the thickening of the INL has been linked to retinal displacement induced by the contraction of the ERM^33^. Consequently, the increased thickness of the INL may clarified the stereoscopic impairment observed in IERM, possibly attributed to retinal misregistration resulting from macular contraction. Besides, studies have underscored the significance of heightened INL thickness in causing visual distortions in unilateral IERM. This is attributed to disruptions in synaptic connections among Muller cells, amacrine cells, bipolar cells, and horizontal cells within the INL, leading to optical signal transmission errors and consequent horizontal and vertical visual deformations^34–37^. Concurrently, the severity of metamorphopsia exhibits a substantial correlation with postoperative TNO value^16^, consist with our findings, that more severe metamorphopsia is an independent prognostic factor for poorer postoperative TNO value. Therefore, the collective influence of structural abnormalities in the macular area and metamorphopsia contributes jointly to the deficits in stereopsis in IERMs.

EIFL, refers to a pseudo inner layer forms in the central fovea due to the extension of the peripheral retina toward the fovea. Normally, the central fovea lacks an inner layer, so its appearance indicates the inward pulling of the retina caused by an epiretinal membrane, suggesting substantial retinal damage. Recently, EIFL status has become recognized as a sensitive and adverse marker for both anatomical recovery and the BCVA prognosis in eyes affected by ERM^38^. However, no study has yet illustrated the direct relationship between EIFL thickness and stereopsis in IERM. Our observations primarily suggested that thinner EIFL correlates with better stereopsis before and after surgery, although these correlations did not reach statistical significance in multivariate regression analyses. The interplay between EIFL and stereopsis might be intricate and influenced by multiple interacting factors, and it is not captured within the scope of this study, implying the need for further research exploring various structural and functional parameters in relation to stereopsis in IERM cases.

The ELM exhibited restored integrity, while the outer photoreceptor lines (COST and EZ band) remained disrupted after surgery in this study, which may be explained by that prolonged inward macular traction initially disrupts the outer segment of photoreceptor cells irreversibly^39,40^. In outer retinopathies and retinal dystrophies, the destruction of visual pigment discs crucial for phototransduction affects photoreceptor function, leading to reduced visual acuity^39,40^. Despite the statistical insignificance in the univariate correlation analysis, the preoperative disruption of the COST band demonstrated a negative association with postoperative stereopsis in the multivariate regression analysis. This association might be explained by affected photoreceptors leading to diminished visual acuity, consequently resulting in a decline in stereopsis.

The superior TST stereopsis and reduced metamorphopsia preoperatively were conspicuously correlated with the improved TST and TNO stereopsis postoperatively in multiple regression analysis. These results underscored the critical importance of preoperative conditions and early intervention for effective functional recovery. Timely surgical intervention might potentially prevent further impairment of stereopsis. Neither TST nor TNO values were linked to the duration or progression of the condition, while a connection between the duration of aggravation and the severity of metamorphopsia was observed and a worse BCVA was reported to be associated with longer duration^18^. The complexity of stereopsis, involving factors such as visual acuity, visual distortion, and differences between affected and healthy eyes, might hinder a direct reflection of duration or aggravated duration similar to BCVA and metamorphopsia. Moreover, the univariate correlation analysis revealed no significant association between surgical procedure and stereopsis, indicating that the impact of PPV combined with cataract surgery on stereopsis might not differ from that of PPV alone.

One significant contributing factor is age. Both preoperative and postoperative stereopsis showed a declined trend as patients aged, suggesting that postoperative recovery of stereopsis in IERM might be limited by age-related factors. According to cross-sectional studies on stereopsis, between 82 and 86% of people have good or normal stereopsis (≤ 60 arc seconds) ^41–43^, and stereopsis undergoes significant changes with age. The physiological deterioration in stereopsis among elderly individuals may stem from age-related cataracts, decreased accommodative abilities, refractive inconsistencies, and declining visual acuity ^41–43^.

In this study, while TNO stereopsis often show marginal improvement, most patients exhibit an enhancement from stereo-blindness to good TST stereopsis. This incongruity might be attributed to different nature between contour-based images in TST and random-dot stereograms in TNO^44,45^. Consequently, the TNO test possesses higher sensitivity and accuracy, making easier binocular separation and fusion challenges, hence a higher likelihood of false-positive results ^22^. Besides, during the Titmus test, patients may rely on the lateral eye to perceive depth (referred to as monocular cues). However, in our study, covering one eye during the TST did not alter the values, suggesting that monocular cues might not strongly affect the outcomes of stereopsis in IERMs. Although, there are differences in the extreme values and levels of these two tests, resulting in a decrease in the comparability of results obtained by the two different examination methods.

The strengths of our study lie in its frequent follow-up within a cohort study, which can also be considered as a before-and-after surgical comparison. To mitigate the potential influence of artificial lenses on stereopsis and to depict an accurate status of the IERM, none of the eyes had a history of lens surgery at the study's outset. Moreover, all surgeries were conducted at a single center by one retina surgeon, ensuring consistency in technique. The evaluation using SD-OCT was independently performed by a single retinal specialist. Limitations include the retrospective nature of our study and relatively small sample. Larger sample sizes or a more homogeneous study population could reveal more conclusive results. Finally, because SD-OCT lacks the capacity to segment individual retinal layers, we drew inspiration from methodologies employed by previous scholars ^21,46^. We opted to use pixel values as a proxy for retinal thickness, while other studies have relied on ImageJ software or customized segmentation systems for this purpose. All these methods entailed manual operations, thereby introducing potential errors. Therefore, resolving technical limitations in OCT is imperative to facilitate more precise and comprehensive research.

## Methods

### Study participants

Fifty-six consecutive patients with unilateral IERM who underwent 25-gauge PPV at the Second Affiliated Hospital of Jilin University between September 2021 and March 2023 were included. The Medical Ethics Committee of the Second Affiliated Hospital of Jilin University approved this retrospective observational study (approval number: 2023-036, approval date: March 30, 2023) performed according to the principles of the Declaration of Helsinki. Written informed consent was obtained from each subject.

Patients with macular hole, diabetic retinopathy, glaucoma, hypertensive retinopathy (retinal vein occlusion), ocular trauma, any specific or non-specific inflammation in the eye (uveitis, phlebitis, retinitis, or endophthalmitis), bilateral ERMs, or a history of lens extraction with IOL implantation, vitrectomy, keratoconus, post-refractive surgery, vitreous hemorrhage, or retinal detachment were excluded. The study also excluded patients with a prior history of strabismus, fusion disorder, or diplopia. Comprehensive ophthalmologic examinations of all the affected eyes were performed preoperatively and 1 week, 1 month, 3 months postoperatively. So, patients with postoperative follow-up of less than 3 months were also not included. These examinations included BCVA, intraocular pressure (IOP), Metamorphopsia, Titmus Stereo Test (Stereo Optical Co., Inc., Chicago, IL, USA and Canada), TNO stereotest (The latest edition of the stereoscopic acuity test of the Netherlands; Netherlands Organisation for Applied Scientific Research), spectral domain OCT (SD-OCT), and slit-lamp microscopy measurements. BCVA was assessed using the international standard visual acuity chart after the patient received the correct prescription. IOP was measured using a non-contact tonometer (NT-510; NIDEK Co., Aichi, Japan). Stereopsis was evaluated at 40 cm and assessed in arc seconds (‘’). Titmus stereo test (with polarized spectacles) measures stereopsis at 2000″ (butterfly upper wing), 1100″ (butterfly antennae), 600″ (butterfly abdomen), 400″, 200″, 160″, 100″, 63″, 50″, 40″, 32″, 25″ and 20″ ^47^. TNO test (with red-green spectacles) measures stereopsis at 480″, 240″, 120″ and 60″, and if the patient failed the TNO quantitative test but passed the qualitative screening, it was recorded as 1980″. If patients failed the Titmus or stereopsis test, 3000″ (gross stereopsis) value was considered for statistical analysis. The patient was instructed to sequentially occlude each eye to assess the monocular cue induced by Titmus stereo test. Subsequently, they were asked if they could perceive the stereoscopic visual beacon, and their response was recorded. The study assessed patients' metamorphopsia with M-CHARTS charts (Inami Co. of Tokyo, Japan: Type I single-line M-CHARTS). Under standard illumination, the chart was placed 30 cm in front of the affected eye at equal height, covering the contralateral eye. The lines on the chart were placed perpendicular to the ground and were identified as curved or not starting with the line at 0°. The value corresponding to the line segment that was first perceived as non-curved was recorded as the vertical metamorphopsia (MV) value. Then the M-CHARTS chart was rotated 90° to align the line segments parallel to the ground, and the value corresponding to the first time the line segments were considered non-bent was noted as the horizontal metamorphopsia (MH) value.

**Figure 4:**
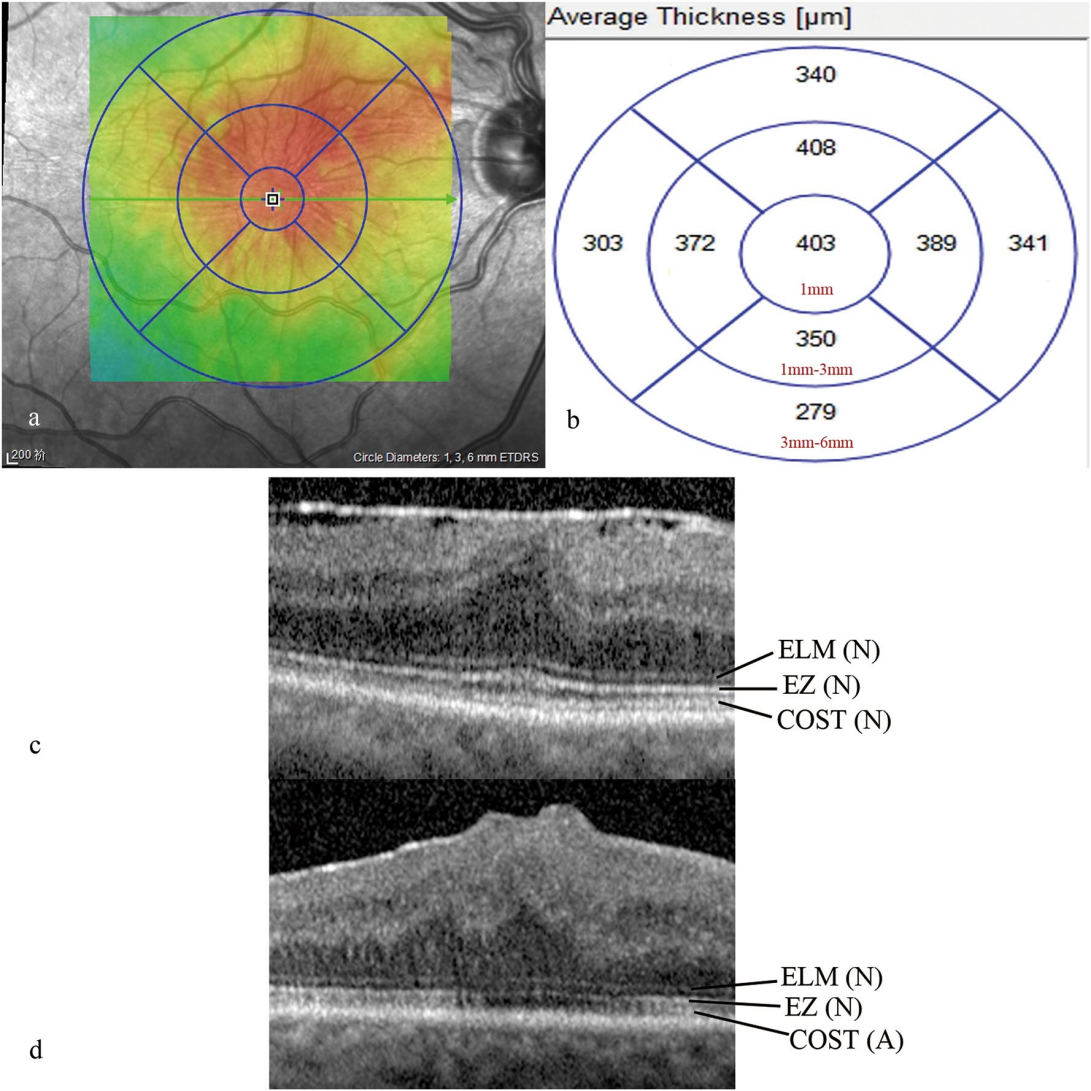
Central retinal thickness and status of the outer retina. **(a)** The macular thickness map automatically divided the central 6 mm × 6 mm image into nine subfields based on the Early Treatment Diabetic Retinopathy Study (ETDRS) grid, **(b)** the mean thickness of the foveal region (1 × 1 mm) represents as the CFT, the average thickness of a circular area with diameters ranging from 1 × 1 mm to 3 × 3 mm as CRT-3 mm, and the average thickness of a circular area with diameters ranging from 3 × 3 mm to 6 × 6 mm as CRT-6 mm. The value in the inner circle represents as the CFT, with value in the middle ring as CRT-3 mm, and the outer ring value as the CRT-6 mm. The average CFT, CRT-3 mm, and CRT-6 mm were 403 mm, 379.75 [(372 + 408 + 389 + 350)/4] mm, and 315.75 [(340 + 341 + 279 + 303)/4] mm, respectively, **(c)** Three highly reflective bands of the outer retina, namely the external limiting membrane (ELM), ellipsoidal band (EZ), and cone outer segment tip (COST) lines. ‘N’ is ‘normal’. The normal status of these bands was characterized by their intact and smooth appearance, **(d)** ‘A’ is ‘abnormal’. A dark area missing in the third highly reflective band of the outer retina means the COST band is abnormal.

**Figure 5:**
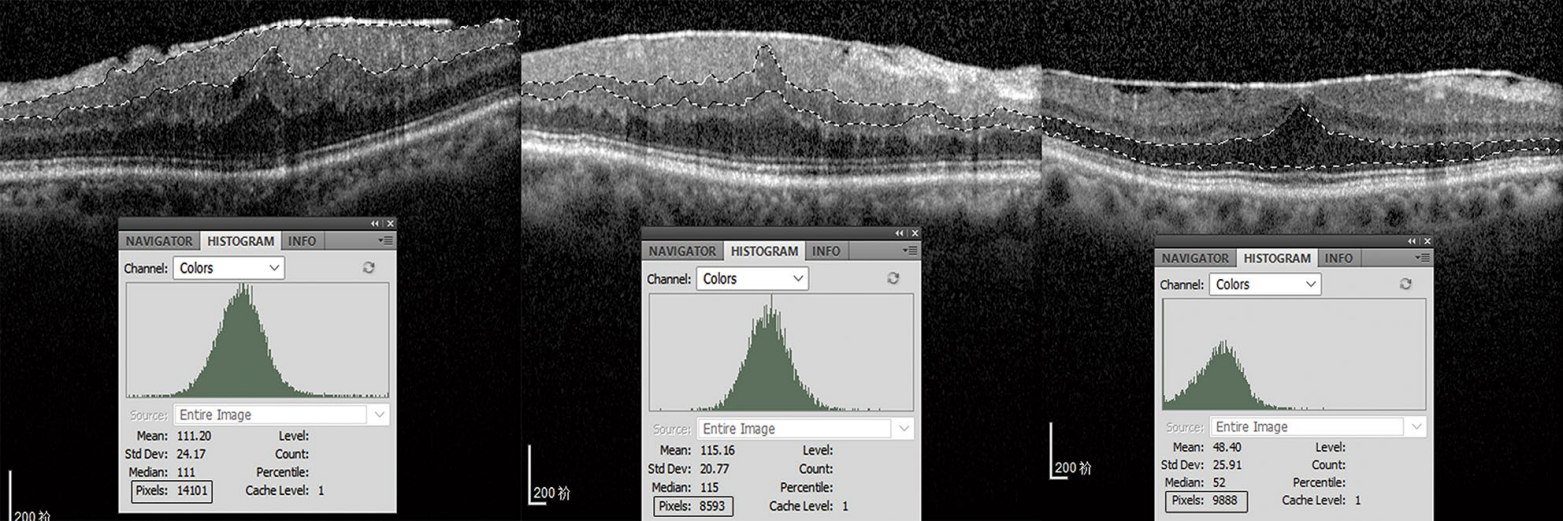
Retinal layers’ thickness. **(a)** The retina was segmented and the color histograms of retinal layers were displayed. The pixel value was shown in the histogram. The ganglion cell layer-inner plexiform layer thickness was 14,101 pixels, **(b)** the inner nuclear layer thickness was 8593 pixels, **(c)** the outer nuclear layer thickness was 9888 pixels.

Participants underwent evaluations of CFT, CRT-3 mm, CRT-6 mm, EIFL thickness, GCL-IPL thickness, INL thickness, ONL thickness and the integrity of photoreceptor high-reflective layers in both eyes. All microstructures were imaged using SD-OCT (Heidelberg Engineering, Heidelberg, Germany). The macular thickness map automatically divided the central 6 mm × 6 mm image into nine subfields based on the Early Treatment Diabetic Retinopathy Study (ETDRS) grid, and the macular thickness in each subfield was measured. The mean thickness of the foveal region (1 × 1 mm) represents as the CFT, the average thickness of a circular area with diameters ranging from 1 × 1 mm to 3 × 3 mm as CRT-3 mm, and the average thickness of a circular area with diameters ranging from 3 × 3 mm to 6 × 6 mm as CRT-6 mm (Fig. 4a, b). The EIFL refers to a continuous band covering the fovea and extending from the inner nuclear and inner plexiform layers surrounding the fovea centralis ^38^. The EIFL thickness was measured manually using the caliper function of the Heidelberg Spectralis platform (Heidelberg Engineering GmbH, Heidelberg, Germany). It was measured as the distance from the inner border of the outer nuclear layer to the inner margin of the internal limiting membrane preoperatively, and from the outer border of the inner nuclear layer to the vitreoretinal interface postoperatively. The ELM, EZ, and COST are three highly reflective lines of the outer photoreceptor layers. The status of these lines was graded as normal when they appeared continuous in the foveal region and as abnormal when disrupted or absent (Fig. 4c, d). The GCL-IPL thickness includes the width of ganglion cell layer and inner plexiform layer. The INL thickness is defined as the width of the inner hypo-reflective band between the outer boundary of the inner plexiform layer and the inner edge of the outer plexiform layer. The retina was segmented by the magnetic lasso tool of Adobe Photoshop CS5 (Adobe Systems, San Jose, CA), and the color histograms of the GCL-IPL, INL, and ONL layer were displayed. Similar to the prior study ^21,46^, we designate the respective pixel values as the thickness of each retina layer (Fig. 5).

All surgeries were performed by a specialist surgeon (J.Z.). The Alcon Constellation/Accurus Vitreoretinal Surgery System and its supporting 25G phacoemulsification heads, perfusion cannula and cannula packages, light guides, etc., as well as the CARL-Zeiss Surgical Microscope were used for the surgical procedure. The surgical technique used was a standard 25G three-port PPV. The ERM was engaged with microscopic forceps after resolving the posterior vitreous detachment (cutting off and aspirating the vitreous gel and posterior hyaloid) and performing core vitrectomy. The inner limiting membrane was not cleared, and a balanced salt solution was used during the procedure. We performed a peripheral retinal examination for scleral depression to identify retinal tears. Patients with retinal tears or detachments in the peripheral retina were excluded. Cataract surgery depends on the degree of lens opacity. When the nuclear turbidity of eded rigidity III (Emery grading standards), phacoemulsification surgery was combined with vitrectomy. Next, the surgeon implanted an intraocular lens into the anterior chamber.

### Statistical analyses

For statistical analysis, the decimal BCVA values were converted into logarithm of the minimal angle of resolution (logMAR) values, and the stereopsis values were converted to logarithmic form. The mean scores and standard deviations were calculated for continuous variables, and frequency and proportions for categorical variables. Comparisons between the parameters at each time point were evaluated using the Friedman Non-parametric Repeated Measures ANOVA Test and Nemenyi post-hoc test for continuous variables, or Paired chi-square test (McNemar's test) for categorical variables, and the significance values were adjusted by Bonferroni correction. Calculated the discrepancy (Wilcoxon signed-rank test) between bilateral eyes by subtracting the retinal thickness of the affected eye from that of the contralateral healthy eye, and the results were considered as optional factors. Age, duration, aggravated duration, surgical procedure and eye category were also included as independent variables. Because all data sets belonged to non-normal distribution, the correlation between any two of parameters were analyzed by Spearman rank correlation analysis and the significance values were double-tailed. Finally, multivariate stepwise linear regression analysis was performed on the correlated parameters with stereopsis to analyze the independent factors. A p-value < 0.05 was considered statistically significant and All statistical analyses were performed using SPSS version 26.0 (IBM Corp., Armonk, NY, USA), and statistical figures were drawn using Prism 9.5.0 (GraphPad Software Inc, San Diego, CA, USA).

### Ethics approval

The study was conducted in accordance with the Declaration of Helsinki, and approved by the Ethics Committee of the Second Hospital of Jilin University (Protocol code: 2023–036, Approval date: March 30, 2023).

### Supplementary Information

Supplementary Figure S1.

Supplementary Table S1.

Supplementary Table S2.

## Data Availability

All data generated or analysed during this study are included in this manuscript and its supplementary information files.
