# Supplementary material for: Optical coherence tomography parameters as prognostic factors for stereopsis after vitrectomy for unilateral epiretinal membrane: a cohort study
Source: Sci Rep. 2024 Mar 20;14:6715. doi: 10.1038/s41598-024-57203-x (PMC10954640; doi:10.1038/s41598-024-57203-x)
Supplement: Supplementary file 1 — Supplementary Figure S1. [file 41598_2024_57203_MOESM1_ESM.pdf]

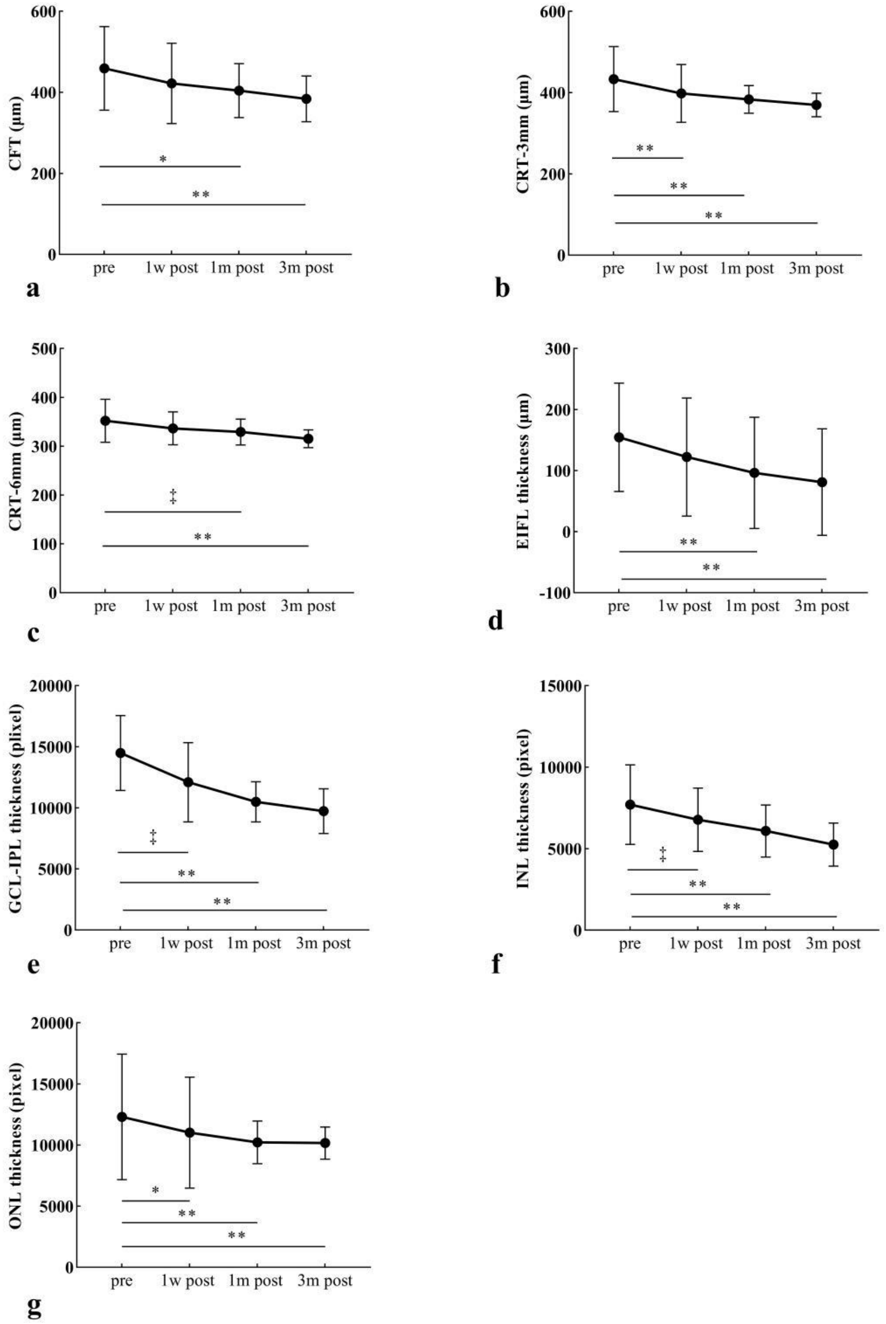

Supplementary Figure 1. Temporal changes of OCT-related parameters in patients with epiretinal membrane before and after surgery. Mean and standard deviation were shown in black dot and error bars. **(a)** Central foveal thickness (CFT). **(b)** Central retinal thickness at the parafovea (CRT-3mm). **(c)** Central retinal thickness at the perifovea (CRT-6mm). **(d)** Ectopic inner foveal layer (EIFL) thickness. **(e)** Ganglion cell layer-inner plexiform layer (GCL-IPL) thickness. **(f)** Inner nuclear layer (INL) thickness. **(g)** Outer nuclear layer (ONL) thickness. Thickness of the central retina and retinal layers significantly thinned after surgery. †$P<0.05$; ‡$P<0.01$; *$P<0.001$; **$P<0.0001$.
