# Supplementary material for: Optical coherence tomography parameters as prognostic factors for stereopsis after vitrectomy for unilateral epiretinal membrane: a cohort study
Source: Sci Rep. 2024 Mar 20;14:6715. doi: 10.1038/s41598-024-57203-x (PMC10954640; doi:10.1038/s41598-024-57203-x)
Supplement: Supplementary file 2 — Supplementary Table S1. [file 41598_2024_57203_MOESM2_ESM.docx]

|  | Titmus Stereo Test | | | | TNO stereotest | | | |
| --- | --- | --- | --- | --- | --- | --- | --- | --- |
|  | Pre | | Post 3months | | Pre | | Post 3months | |
|  | r | p | r | p | r | p | r | p |
| Pre TST |  |  | 0.673 | ﹤0.001* | 0.479 | ﹤0.001* | 0.542 | ﹤0.001* |
| Post 3months TST | 0.673 | ﹤0.001* |  |  | 0.530 | ﹤0.001* | 0.649 | ﹤0.001* |
| Pre TNO | 0.479 | ﹤0.001* | 0.530 | ﹤0.001* |  |  | 0.126 | 0.335 |
| Post 3months TNO | 0.542 | ﹤0.001* | 0.649 | ﹤0.001* | 0.126 | 0.335 |  |  |
| Pre BCVA | 0.410 | 0.002* | 0.209 | 0.122 | 0.176 | 0.194 | 0.275 | 0.040* |
| Post 1month BCVA | 0.283 | 0.035* | 0.466 | ﹤0.001* | 0.247 | 0.067 | 0.470 | ﹤0.001* |
| Post 3months BCVA | 0.354 | 0.008* | 0.444 | 0.001* | 0.122 | 0.372 | 0.543 | ﹤0.001* |
| Pre MV | 0.117 | 0.389 | -0.072 | 0.597 | -0.116 | 0.394 | 0.330 | 0.013* |
| Post 3months MV | -0.010 | 0.942 | 0.112 | 0.410 | -0.138 | 0.312 | 0.383 | 0.004* |
| Pre MH | -0.042 | 0.757 | -0.193 | 0.155 | -0.094 | 0.489 | 0.074 | 0.587 |
| Post 3months MH | -0.048 | 0.725 | 0.063 | 0.642 | 0.084 | 0.538 | 0.035 | 0.796 |

Table S1. Correlation between stereopsis and BCVA, metamorphopsia in patients with ERM.

*Signifcant correlation between parameters (Spearman rank correlation test).

Pre=preoperaive, Post=postoperative, BCVA=best-corrected visual acuity, MV=vertical metamorphopsia, MH=horizontal metamorphopsia. ERM:epiretinal membrane, TST=Titmus Stereo Test, TNO=TNO stereotest.
